# Aortic Dissection in Familial Patients with Autosomal Dominant Polycystic Kidney Disease

**DOI:** 10.3400/avd.cr.20-00149

**Published:** 2021-03-25

**Authors:** Yu Inaba, Motohiko Osako, Michiko Aoki, Mio Kasai, Kentaro Yamabe

**Affiliations:** 1 Department of Cardiovascular Surgery, National Hospital Organization Tokyo Medical Center, Tokyo, Japan

**Keywords:** aortic dissection, ADPKD

## Abstract

Autosomal dominant polycystic kidney disease (ADPKD) is the most common congenital kidney disease. However, reports on occasional cases of aortic dissection in PKD familial patients remain scarce. Herein, we describe rare aortic dissection cases in PKD familial patients (i.e., mother and daughter) and our successful treatment experience. The mother (84 years old) and daughter (53 years old) had a referral to us to treat type A acute aortic dissection. We performed emergency surgery and successfully treated the patients with an artificial graft. For comprehensive evaluation and treatment, ADPKD patients and their families should be screened for aortic diseases.

## Introduction

Autosomal dominant polycystic kidney disease (ADPKD) is the most common congenital kidney disease. ADPKD is genetically heterogeneous, and mutation of the polycystic kidney disease (PKD) 1 and PKD 2 genes contributes to its development. The frequency of intracranial aneurysms in ADPKD patients is reportedly 9%–12%.^1)^ However, there are few reports on occasional cases of aortic dissection in PKD familial patients. Herein, we report aortic dissection cases in PKD familial patients (i.e., mother and daughter) and our successful treatment experience.

## Case Reports

**Figure 1:**
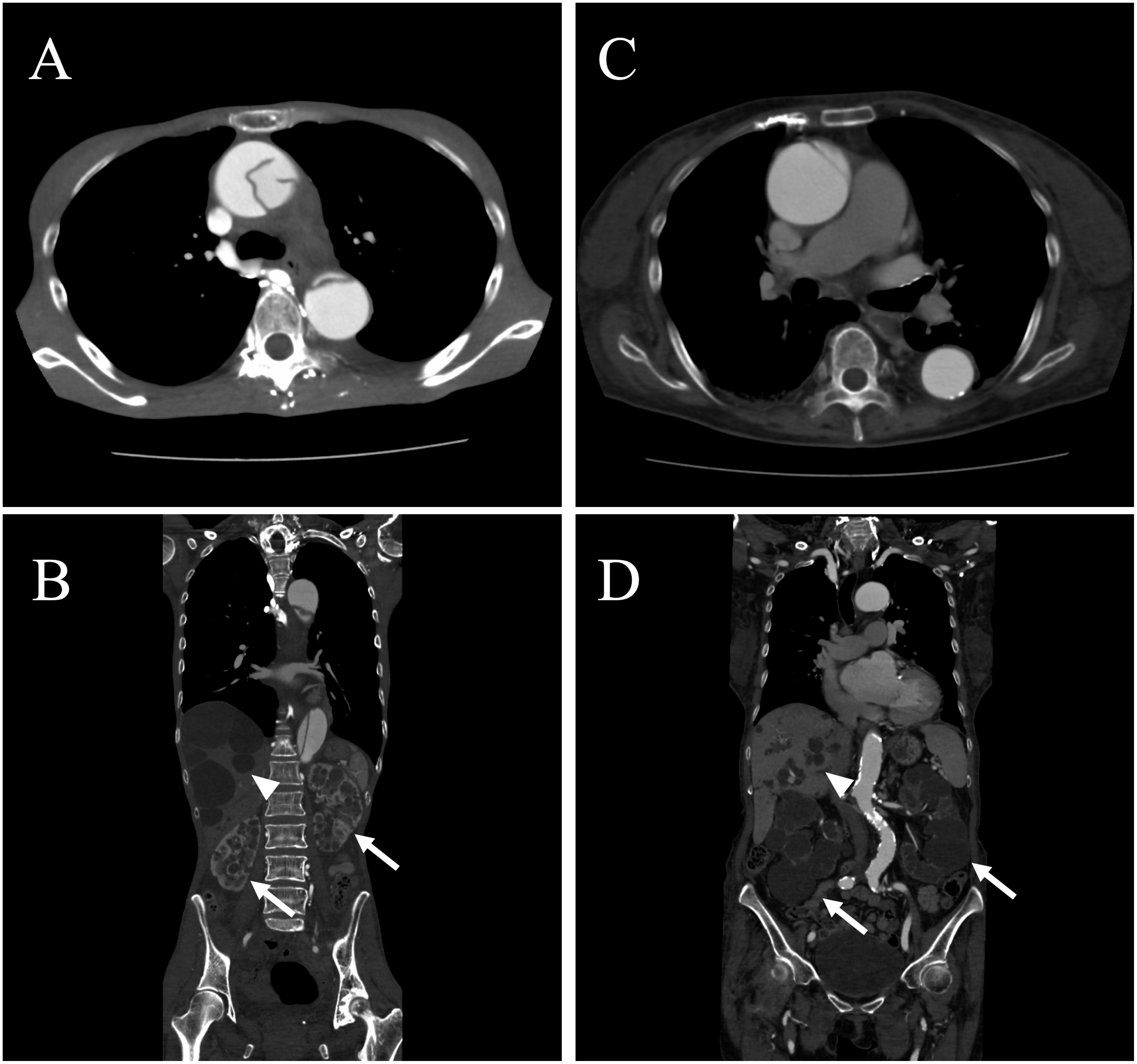
Fig. 1 Computed tomography of patients. Multiple renal cysts (white arrows) and liver cysts (white arrowheads) were detected. (**A**) Axial view of the daughter. (**B**) Coronal view of the daughter. A polycystic kidney was detected (white arrows). (**C**) Axial view of the mother. (**D**) Coronal view of the mother. A polycystic kidney was detected (white arrows).

**Figure 2:**
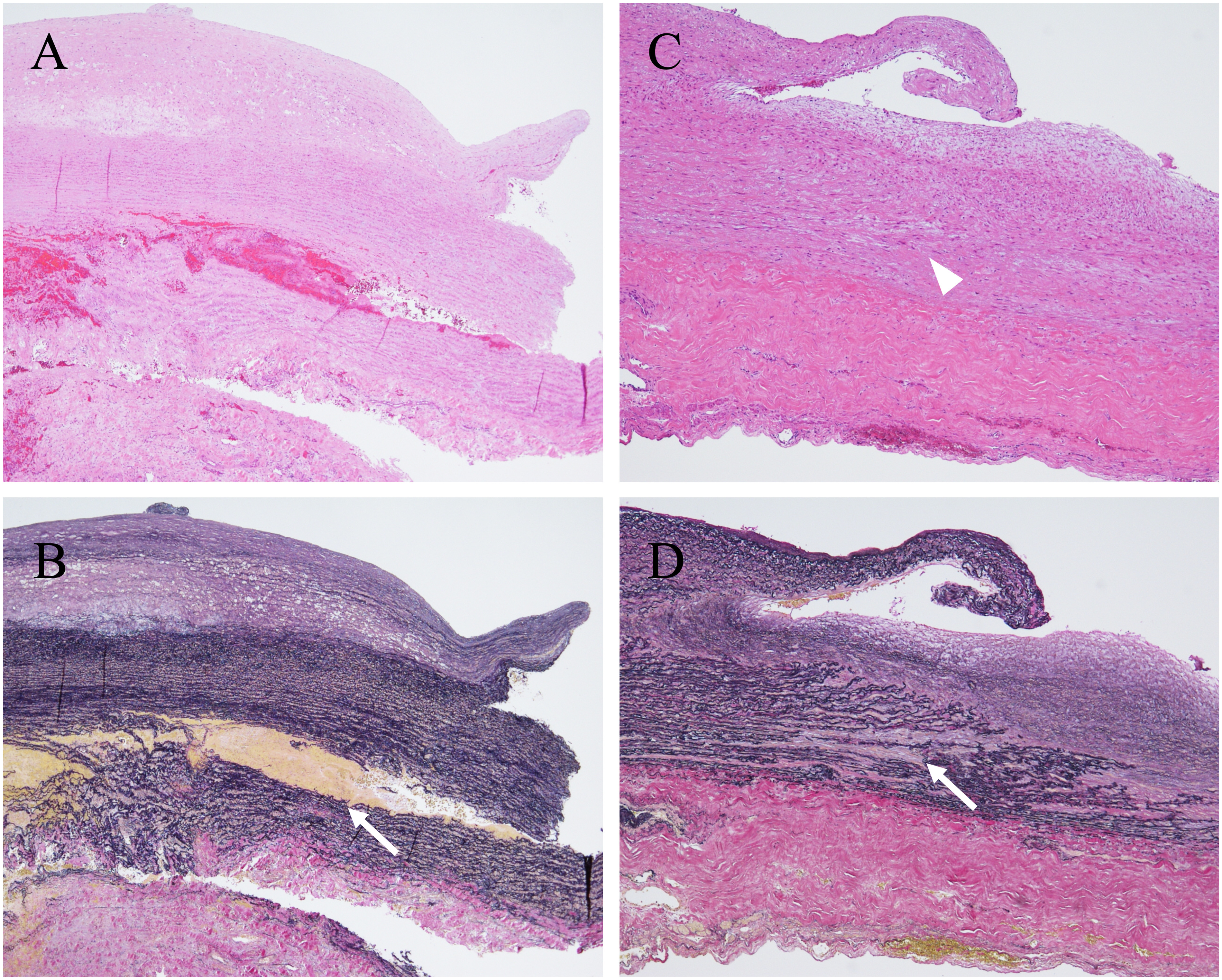
Fig. 2 Histological analysis of the daughter (**A**, **B**) and mother (**C**, **D**). (**A**) and (**C**): hematoxylin/eosin staining. (**B**) and (**D**): Elastica van Gieson staining. A rupture of elastic fibers was detected in these patients (white arrows). Cystic medial necrosis was detected in the mother’s aortic tissue (white arrowhead).

A 53-year-old woman came to the emergency department for a sudden onset of back pain. The patient had a medically managed hypertension from five years ago. Computed tomography (CT) showed acute type B aortic dissection, polycystic kidney, and multiple hepatic cysts. Blood tests on admission revealed a creatinine level of 0.95 mg/dl and an estimated glomerular filtration rate (eGFR) of 48.5 ml/min/1.73 m^2^. The patient received a referral to our center for medical treatment. Sixteen days later, the patient suffered from severe chest pain. A CT showed acute type A aortic dissection (**Figs. 1A** and **1B**), and the patient underwent emergency surgery. A dissection entry was detected from the ascending aorta to the distal aorta. The patient underwent a total aortic arch replacement with a 3-branched artificial graft (J-Graft, Japan Lifeline, Tokyo, Japan) and a Frozen elephant trunk (FROZENIX, Japan Lifeline). She did not require postoperative hemodialysis. The patient was discharged one month postoperatively. Histological analysis showed rupture of the internal elastic lamina and tunica media and inflow of blood. There was a high degree of atherosclerosis in the tunica intima and media. Elastica van Gieson staining showed no medial cystic necrosis (**Figs. 2A** and **2B**).

One week from discharge, the 84-year-old patient’s mother was referred by another hospital to our center for type A aortic dissection. CT showed type A aortic dissection, polycystic kidney, and multiple hepatic cysts (**Figs. 1C** and **1D**). A dissection entry was found in the ascending aorta. Blood tests on admission revealed a creatinine level of 3.82 mg/dl and an eGFR of 9.3 ml/min/1.73 m^2^. The patient had medically managed hypertension from 50 years of age with a history of PKD and renal failure. An urgent ascending aortic replacement was performed using a straight artificial graft (J-Graft, Japan Lifeline). Although the patient required transient hemodialysis in the intensive care unit, hit was unnecessary at discharge. The patient was moved to another hospital for rehabilitation one month postoperatively. Histological analysis showed aortic dissection, rupture of elastic fibers, and medial cystic necrosis (**Figs. 2C** and **2D**). The postoperative magnetic resonance imaging showed no cerebral aneurysms in both patients. Her brother had died of aortic dissection several years earlier, but his detailed medical history was unknown.

## Discussion

ADPKD is the most common congenital renal cystic disease with an estimated prevalence of between one in 1000 and one in 2500 individuals.^1)^ Its course is characterized by the development and inexorable expansion of multiple cysts scattered throughout the kidney parenchyma. ADPKD patients have a 10% incidence of intracranial aneurysms.^2)^ Mitral valve prolapse occurs in up to 26% of PKD-1 patients.^3)^ Aortic dissection is a rare complication of ADPKD, and there are few reports on the frequencies of aortic aneurysm and aortic dissection. ADPKD is genetically heterogeneous, and the PKD 1 and PKD 2 genes’ mutation contributes to its development. Mutations in PKD genes that encode polycystin, which is often expressed in vascular smooth muscle, including the kidney, are suggested to cause comorbid cysts and cardiovascular abnormalities. In a mouse model research, PKD 1 has been implicated in maintaining vessel wall structural integrity.^4)^

This is a rare report of aortic dissection in ADPKD familial patients successfully repaired with an artificial graft to the best of our knowledge. The congenital polycystic kidney and multiple renal cysts detected by CT fulfill the ADPKD criteria of Ravine et al.^5)^ An interesting finding is that the three family members similarly suffered from an aortic dissection. The reason for the histological difference between the mother and the daughter is unclear. Although medial cystic necrosis appeared in the mother’s aortic wall, it was undetected in the daughter. Cystic medial necrosis may not necessarily cause aortic dissection. Other factors, including changes in the extracellular matrix or cellular interaction from PKD gene mutation, may cause aortic dissection.

The mother and daughter were diagnosed with ADPKD before the onset of acute aortic dissection (AAD), implying an important finding that the cause of their AAD was ADPKD. Hypertension and renal failure associated with ADPKD were considered significant risk factors for aortic dissection following arteriosclerosis.^6)^ A systematic review described a markedly higher frequency of hypertension and younger age in aortic dissection patients with ADPKD than the overall aortic dissection population. The earlier aortic dissection manifestation and the possible lack of symptoms suggestive of aortic dissection in ADPKD patients underline the importance of performing close clinical screening from a young age.^6)^ Antihypertensive therapy may prevent renal failure in ADPKD patients.^7)^

Regarding the emergency treatment of acute aortic dissection, antihypertensive therapy is crucial for preventing the progress of the dissection and organ malperfusion. The renin angiotensin system’s activity is reportedly increased in ADPKD. Antihypertensive therapy using an angiotensin-converting-enzyme inhibitor may be effective in acute aortic dissection patients with ADPKD.^8)^

In conclusion, we successfully treated acute type A aortic dissection in ADPKD familial patients. For comprehensive evaluation and treatment, ADPKD patients and their families should be screened regularly for aortic diseases.
